# Cancer testis antigens and NY-BR-1 expression in primary breast cancer: prognostic and therapeutic implications

**DOI:** 10.1186/1471-2407-13-271

**Published:** 2013-06-03

**Authors:** Dimitrios Balafoutas, Axel zur Hausen, Sebastian Mayer, Marc Hirschfeld, Markus Jaeger, Dominik Denschlag, Gerald Gitsch, Achim Jungbluth, Elmar Stickeler

**Affiliations:** 1 Department of Obstetrics and Gynecology, University Hospital Freiburg, Hugstetterstraße 55, Freiburg 79106, Germany; 2 Department of Pathology, GROW- School for Oncology and Developmental Biology, Maastricht University Medical Center, Postbus 5800, Maastricht 6202 AZ, The Netherlands; 3 German Cancer Consortium (DKTK), Heidelberg, Germany; 4 German Cancer Research Center (DKFZ), Heidelberg, Germany; 5 Ludwig Institute for Cancer Research, New York Branch at Memorial Sloan-Kettering Cancer Center, 1275 York Avenue, BOX 32, New York, NY 10021-6007, USA

**Keywords:** Breast Cancer, Cancer-testis Antigen, NY-BR-1, Immunotherapy, Prognosis

## Abstract

**Background:**

Cancer–testis antigens (CTA) comprise a family of proteins, which are physiologically expressed in adult human tissues solely in testicular germ cells and occasionally placenta. However, CTA expression has been reported in various malignancies. CTAs have been identified by their ability to elicit autologous cellular and or serological immune responses, and are considered potential targets for cancer immunotherapy. The breast differentiation antigen NY-BR-1, expressed specifically in normal and malignant breast tissue, has also immunogenic properties. Here we evaluated the expression patterns of CTAs and NY-BR-1 in breast cancer in correlation to clinico-pathological parameters in order to determine their possible impact as prognostic factors.

**Methods:**

The reactivity pattern of various mAbs (6C1, MA454, M3H67, 57B, E978, GAGE #26 and NY-BR-1 #5) were assessed by immunohistochemistry in a tissue micro array series of 210 randomly selected primary invasive breast cancers in order to study the diversity of different CTAs (e.g. MAGE-A, NY-ESO-1, GAGE) and NY-BR-1. These expression data were correlated to clinico-pathological parameters and outcome data including disease-free and overall survival.

**Results:**

Expression of at least one CTA was detectable in the cytoplasm of tumor cells in 37.2% of the cases. NY-BR-1 expression was found in 46.6% of tumors, respectively. Overall, CTA expression seemed to be linked to adverse prognosis and M3H67 immunoreactivity specifically was significantly correlated to shorter overall and disease-free survival (p=0.000 and 0.024, respectively).

**Conclusions:**

Our findings suggest that M3H67 immunoreactivity could serve as potential prognostic marker in primary breast cancer patients. The exclusive expression of CTAs in tumor tissues as well as the frequent expression of NY-BR-1 could define new targets for specific breast cancer therapies.

## Background

Breast cancer is the second most common human malignancy [1]. In recent years the progress in systemic treatment modalities, especially endocrinological, immuno- and chemotherapeutical strategies, have substantially reduced the proportion of women who develop metastatic disease. In the context of these advances the importance to identify prognostic and predictive markers is steadily increasing in order to avoid unnecessary adjuvant therapy regimens [2].

Cancer testis antigens (CTAs) comprise an expanding family of proteins which are normally expressed in human testicular germ cells or placental trophoblast, but not in any other normal tissue. However, CTAs are present in various malignancies [3]. More than 100 CTA-related genes and/or gene families have been identified, however their biological function remains poorly understood. CTA encoding genes which are located on chromosome X are referred to as CT-X antigens. Expression of these antigens has been found in diverse malignant human tumors including breast cancer [4]. Because of their restricted expression, CTAs are considered relevant to cancer biology and their prognostic relevance has been assessed in the recent years by several studies for various malignancies [5,6]. Yet the prognostic significance of CTAs in breast cancer still remains unclear.

Interestingly the presence of some CTAs such as, MAGE-A family members, GAGE and NY-ESO-1 appears to correlate with clinico-pathological parameters and prognosis in tumors, such as melanoma, non-small-cell lung cancer, multiple myeloma and other tumors [7]. CTAs are frequently recognized by cytotoxic T-lymphocytes of cancer patients or they can elicit a serological immune response in the autologous host [8]. Consequently, CTAs are regarded potential candidates for the development of anti-cancer vaccines [9,10]. Specifically NY-ESO-1 is able to elicit combined humoral and cell mediated immune response and considered to be the most immunogenic of the above antigens. Therefore NY-ESO-1 based vaccines have been employed in several clinical vaccination trials [11].

NY-BR-1 is a differentiation antigen of the mammary tissue, since it has been detected solely in the epithelial cells of mammary ducts and lobules, whereas NY-BR-1 expression has not been found in any other tissue [12]. Thus, NY-BR-1 appears to be a breast-specific protein.

At present only few reports on CTA expression patterns and their prognostic role in breast cancer are available with limited number of patients and clinical correlations and in part controversial findings [4,13-18]. The objective of this study was to examine the expression pattern of the aforementioned CT-antigens as well as NY-BR-1 in breast cancer and to correlate them with clinico-pathological parameters including patient outcome data. This study is the first to analyze simultaneously the expression of the CTAs and NY-BR-1 in a patient collective with long-term follow up data.

## Methods

### Patients

For this study 210 consecutive patients diagnosed with invasive breast cancer were enrolled, according to the ethics committee of the University Hospital Freiburg, Germany (EK-Freiburg 324/09). Standard archival paraffin blocks of primary breast cancer were retrieved from the archives of the Department of Pathology of the University Hospital Freiburg. All patients underwent surgery in the Breast Unit of the Department of Gynecology of the University Hospital Freiburg. Primary treatment consisted of radical mastectomy, modified radical mastectomy, or breast-conserving surgery including sentinel and/or axillary lymph node dissection between the years 1991 and 2001. Patients who received neoadjuvant chemotherapy, or who underwent preceding treatment at another institution or patients with a second primary tumor were excluded. Median age at the time of diagnosis was 57 years. Histopathological analyses demonstrated invasive ductal cancer in 73.8% of cases and invasive lobular subtype in 7.6%. The remaining 18.6% were diagnosed as ductal/lobular, mucinous (colloid), tubular, medullary and papillary carcinomas, respectively. In 88/210 (41.9%) patients lymph node involvement was histologically confirmed at the time of surgery. 146/210 (69.5%) of the tumors were estrogen or progesterone receptor positive. Immunohistochemical Her2/neu overexpression was recorded in 40 (21.2%) of the cases.

Follow up ranged from 1 to 107 months (mean 62, median 68 months), recurrences occurred in 59 (28.1%) and deaths in 43 (20.5%) of women, respectively. The 63 cases with technical failure in microarray mapping were excluded from the study.

### Materials

Paraffin-embedded tissue blocks were used to generate tissue-microarrays (TMAs). At least three representative cores of each tumor were selected. Two specimens of normal breast as well as non neoplastic breast tissue adjacent to the lesions were used as controls. Four micron paraffin sections were stained immunohistochemically as previously described [16]. The following monoclonal antibodies (mAbs) were used: mAb 6C1 (Santa Cruz Biotechnology, Inc., Santa Cruz, USA) to several members of the MAGE-A family, mAb MA454 to MAGE-A1, mAb M3H67 also to several members of the MAGE-A family and mAb 57B to MAGE-A4 [19-21]. Next to these, the immunoreactivity of mAb E978 to NY-ESO-1 [22] and mAb #26 (BD Biosciences Clontech, Palo Alto, USA) to GAGE was assessed. For the detection of NY-BR-1, mAb NY-BR-1#5 previously generated by our group was utilized [23].

Evaluation of the immunohistochemical staining was performed in a blinded set up regarding the clinical data. Scoring of the expression was performed semiquantitatively as described previously [24]. In brief, both percentage of stained cells and staining intensity were evaluated. No staining or weak staining in <5% of cells was defined as 0, weak staining in at least 5% as 1, moderate staining in up to 50% as 2 and moderate staining in >50% of cells and strong staining of any percentage of the cells as 3. The results were subsequently dichotomized for statistical analysis and the defined cut-off point for positivity for the statistical analysis was set to 2.

Our data were analysed using the statistical package SPSS for windows version 17.0 (SPSS, Chicago, Illinois, USA). The relationship among clinico-pathological parameters and CTA expression were tested using the chi-square and Fisher’s exact test. Survival outcomes were analysed with Kaplan-Meier survival functions and compared between groups with the log-rank statistics. To determine the association of clinico-pathological parameters with survival, univariate and multivariate Cox regression models were used. The multivariate Cox regression model was adjusted for any known prognostic variables with p<0.05. For all tests p<0.05 was accepted as threshold of statistical significance. Cases with missing microarrays for some of the antigens were handled in the statistical analysis as missing data.

## Results

### Expression of CTAs and NY-BR1 in invasive breast cancer

**Figure 1:**
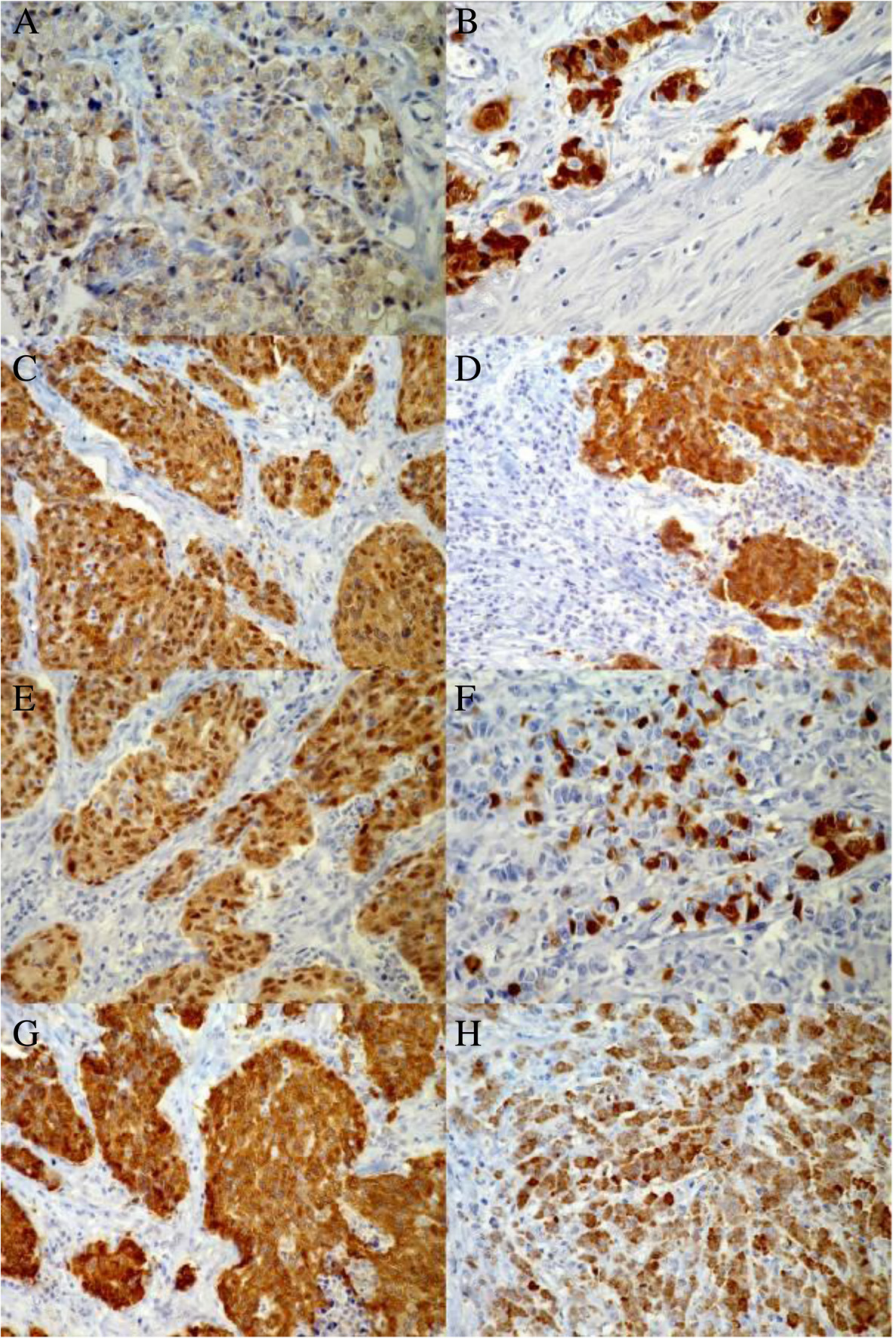
**Immunohistochemical detection of cancer - testis antigens and NY-BR-1 in primary breast cancer tissue microarrays. A**: Example of moderate staining of MAGE A1 in approximately 80% of the tumor cells. The staining is restricted to the cytoplasm. **B**: Strong nuclear and cytoplasmic expression of MAGE A1. **C**: Extensive strong nuclear and cytoplasmic M3H67 immunoreactivity. **D**: Strong, mainly cytoplasmic and occasionally nuclear 57B immunoreactivity. **E**: Extensive strong nuclear and cytoplasmic detection of MAGE A (6C1). **F**: Focal strong, mainly cytoplasmic staining of approximately 20% of tumor cells for GAGE. **G**: Strong extensive cytoplasmic and occasionally nuclear staining of NY-ESO-1. **H**: Strong cytoplasmic NY-BR-1 staining of approximately 80% of cells with scarce nuclear detection (40x objective).

Overall, CTA expression was restricted to neoplastic breast tissues and detected in 54 tumor samples (37.2%) (Table 1). The expression was mainly restricted to the cytoplasm and only occasionally located in the nuclei (Figure 1). A heterogeneous expression pattern was observed regarding the percentage of positive tumor cells.

**Table 1:** Frequency of immunohistochemical detection of CTAs and NY-BR-1 with the corresponding mAbs in breast cancer

	**n**	**%**
MAGE-A1 MA 454	21 (140)	15
mAb M3H67	17 (132)	12,9
mAb 57B	6 (133)	4,5
NY-ESO-1 E978	21 (140)	15
GAGE #26	17 (133)	12,8
MAGE-A 6C1	7 (141)	5
NY-BR-1 #5	61 (131)	46,6

n=number of cases with antigen positivity, in parenthesis total number with successful TMA mapping for each antigen.

MA454 reactivity (MAGE-A1) was found in 21 of cases (15%). In the 14 cases with moderate staining (10.0%) this was restricted solely to the cytoplasm, whereas in the 7 (5.0%) cases with strong staining both the nuclei and the cytoplasm were positive.

E978 reactivity (NY-ESO-1) was also found in 21 of cases (15%). In 15 (10.7%) cases the staining was of moderate intensity with cytoplasmic localisation and in 6 (4.3%) samples it was strong cytoplasmatic with occasional nuclear participation.

M3H67 reactivity was detected in 17 (12.9%) of cases. Moderate staining was observed in 8 (6.1%) and strong staining in 9 (6.8%) of cases. On the cellular level, in the cases of moderate staining the localisation was predominantly cytoplasmic and in the cases of strong staining it was both cytoplasmic and nuclear.

MAb #26 reactivity (GAGE) was detected in 17 (12.8%) of cases analysed. GAGE localisation was primarily cytoplasmic with some rare nuclear participation. Moderate expression was found in 9 (6.8%) and strong expression in 8 (6.0%) of cases.

57B reactivity (MAGE-A4) was found in 6 (4.5%) of the arrays and the staining in these cases was classified as strong. Localisation was cytoplasmic with concomitant nuclear staining in approximately 20% of the nuclei in the positive areas. In 22 (10.5%) of cases we observed a very weak unspecific diffuse cytoplasmic staining which was considered negative in the analyses.

Seven (5.0%) cases revealed mAb 6C1 reactivity. In 3 (2.1%) of cases the staining was moderate, predominately cytoplasmic and to a lesser extend nuclear and in 4 (2.8%) it was strong, with both nuclear and cytoplasmic expression.

The breast differentiation antigen NY-BR-1 was immunohistochemically detected with the #5 Mab in the ductal and lobular cells of all included non neoplastic tissues as well as in 61 of 131 cases of cancer (46.6%). Its expression was predominantly cytoplasmic in the normal and in the tumorous tissue with nuclear participation of varying degree. The staining intensity was classified as moderate in 39 (29.8%) and as strong in 22 (16.8%) of cases. Some areas with dot-like staining pattern in the cytoplasm were also observed. No correlation was found between expression of CTAs and NY-BR-1.

### Correlations with clinico-pathological parameters

The expression data of each CTA were grouped based on clinico-pathological characteristics (Table 2): Age group (in comparison to median), tumor size and grade, lymph node involvement, histological type, estrogen and progesterone receptor and HER2/neu status were compared among positive and negative samples for each CTA. Interestingly, we observed that CTA positivity in our cohort was restricted to grade 2 and 3 tumors and all grade 1 tumor samples were negative for all investigated CTAs. For the other examined parameters there was no significant difference between CTA positive and negative groups.

**Table 2:** Clinicopathological characteristics of breast cancer patients in our collective

		**n (%)**
Age	<median	105(50%)
	>median	105(50%)
Tumor grade	1	7(3,50%)
	2	92(45,80%)
	3	102(50,70%)
Tumor size	pT1	88(46,60%)
	pT2	78(41,30%)
	pT3/4	23(12,20%)
Lymph node status	pN0	119(57,50%)
	pN1/2/3	88(42,50%)
Histological type	ductal	75(58,10%)
	lobular	16(12,40%)
	other	38(29,40%)
ER/PR status	negative	58(28,40%)
	positive	146(71,60%)
HER2/neu	0	54(28,60%)
	1	84(44,40%)
	2	11(5,80%)
	3	40(21,20%)

The expression frequency of NY-BR-1 was equally distributed among the groups with different tumor grading. Similarly we did not find any significant differences in the expression of NY-BR-1 related with other clinicopathological parameters.

### Clinical outcome analysis

Factors associated with disease-free survival (DFS) and disease specific overall survival (OS) were analysed by Univariate Cox regression (Table 3). We observed a statistically significant negative prognostic impact for larger tumor size (p=0.002 for both DFS and OS) and lymph node metastases (p=0.000 for both DFS and OS). The expression of estrogen or progesterone receptor was accompanied by longer DFS (p=0,019), but for OS this correlation did not reach statistical significance. In the univariate Cox regression analysis NY-BR-1 did not seem to affect recurrence or survival.

**Table 3:** Univariate-Cox-regression-analysis of known prognostic factors CTAs and NY-BR-1 of breast-cancer patients

		**DFS**			**OS**	
	**HR**	**95% CI**	**p**	**HR**	**95% CI**	**P**
Age vs median	0,884	0,530–1,475	0,637	0,987	0,542–1,796	0,966
Tumor grade	1,521	0,930–2,488	0,095	1,652	0,903–3,022	0,104
Tumor stage	1,808	1,241–2,632	0,002	1,988	1,292–3,058	0,002
Lymph node status	2,971	1,730–5,104	0	3,348	1,714–6,537	0
Histological type	0,864	0,672–1,111	0,255	0,855	0,624–1,170	0,327
ER/PR status	0,526	0,307–0,901	0,019	0,775	0,399–1,505	0,451
HER2/neu status	1,064	0,826–1,369	0,632	0,986	0,727–1,337	0,929
MAGE A1 MA454	1,278	0,564–2,898	0,557	2,284	0,910–5,732	0,078
M3H67 reactivity	2,85	1,350–6,017	0,006	4,27	1,834–9,941	0,001
57B reactivity	3,406	1,15–10,03	0,026	3,446	1,00–11,77	0,049
NY-ESO-1 E978	1,272	0,563–2,877	0,563	0,805	0,242–2,684	0,724
GAGE #26	1,988	0,875–4,516	0,101	1,98	0,739–5,303	0,174
MAGE A 6C1	1,65	0,498–5,466	0,413	1,876	0,435–8,087	0,399
NY-BR-1 #5	1,522	0,816–2,839	0,186	1,235	0,554–2,752	0,606

Abbreviations: *DFS* Disease free survival; *OS* Overall survival; *HR* Hazard ratio; *CI* Confidence interval.

**Figure 2:**
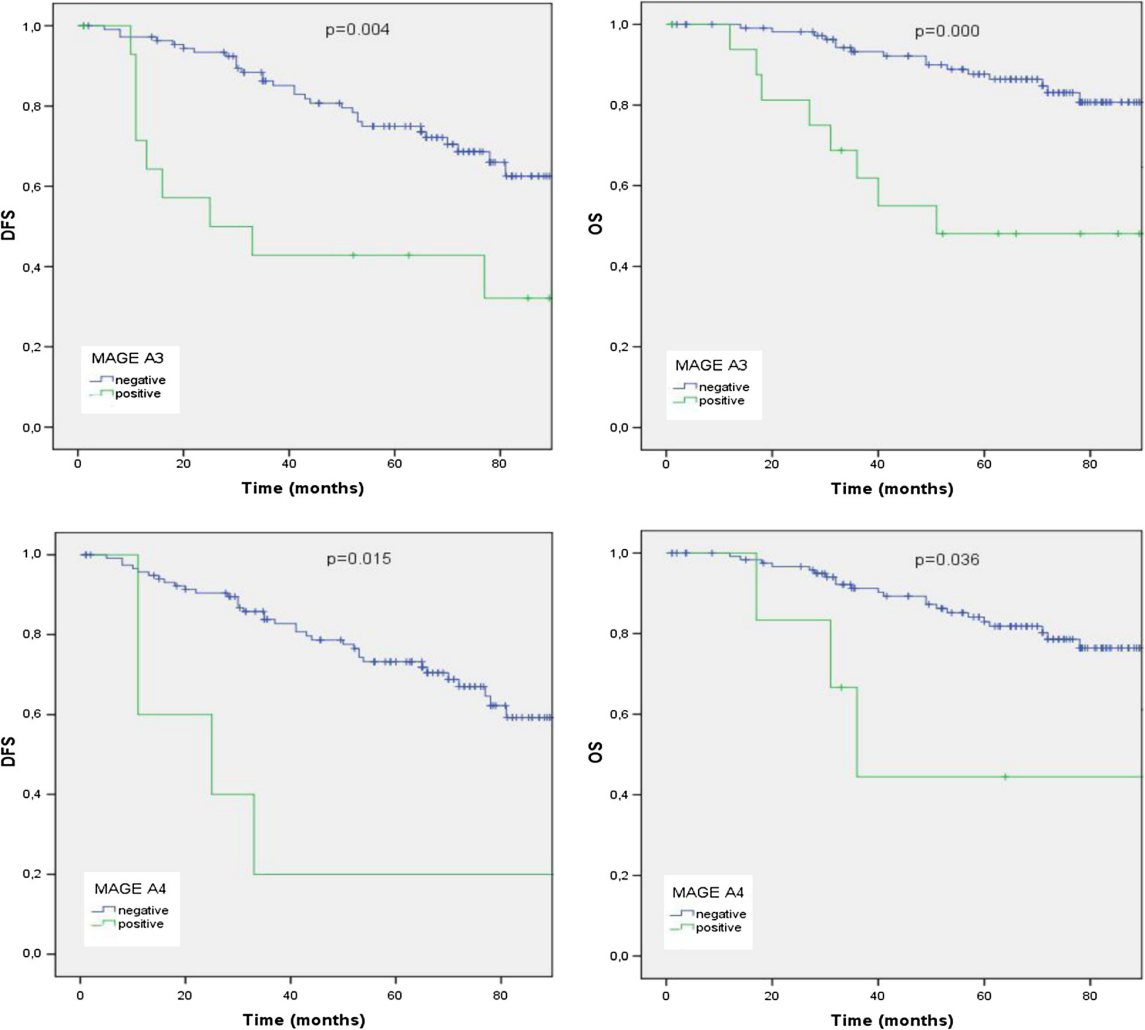
**Kaplan Meier survival analysis for disease-free (DFS) and overall-survival (OS): In the presence (green line) or absence (blue line) of immunohistochemical reactivity of M3H67 and 57B.** p: log rank test.

In contrast to NY-BR-1, Kaplan-Meier survival analysis (Figure 2) demonstrated a strong clinical impact on survival for the immunoreactivity pattern of most of the examined CTAs. The detected adverse effects were statistically significant for both recurrence and disease related death for M3H67 (p-log rank=0.004 and 0.000) and 57B (p-log rank=0.015 and 0.036) immunoreactivity, respectively. MAGE-A1 positive patients had a shorter OS (p-log rank =0.028), but no impact on DFS was observed. Additionally we found a clear, though statistically not significant trend for negative effects of mAb #26 (GAGE) and mAb 6C1 (MAGE-A family) expression on DFS and OS: GAGE positive patients had a 19.9% shorter DFS and a 14.7% shorter OS (p-log rank =0.090 and 0.238) and 6C1 positive patients a 23.19% shorter DFS and a 16.97% shorter OS (p-log rank =0.090 and 0.453).

### Multivariate analysis identifies M3H67 reactivity as a strong prognosticator for overall survival

In order to identify the independent prognostic factors in our cohort we performed a multivariate Cox regression analysis (Table 4). Lymph node status was confirmed as a known independent prognostic parameter with a hazard ratio (HR) 6.37 (95% CI 2.6–17.4, p=0.0001) and 5.99 (95% CI 1.9–18.7, p=0.002) for DFS and OS respectively. However, M3H67 reactivity exhibited the strongest prognostic impact in this study, with a HR of 7.69 (95% CI 2.6–22.8, p=0.0001) for OS and the second strongest for DFS with a HR of 4.36 (95% CI 1.2–15.6, p=0.024). Estrogen or progesterone receptor positivity was correlated with decreased risk of disease recurrence (HR 0.40, 95% CI 0.1–0.8, p=0.015) but was not included in the multivariate analysis for overall survival, because it did not reach the significance threshold in univariate analysis.

**Table 4:** Multivariate-Cox-regression-analysis for disease-free survival and overall-survival of breast-cancer patients

		**DFS**			**OS**	
	**HR**	**95% CI**	**p**	**HR**	**95% CI**	**P**
Tumor stage	0,807	0,435–1,496	0,496	1,393	0,658–2,948	0,386
Lymph node status	6,737	2,607–17,409	0,000	5,99	1,920–18,688	0,002
ER/PR status	0,405	0,196–0,837	0,015			
M3H67 reactivity	4,355	1,218–15,572	0,024	7,693	2,597–22,786	0,000
57B reactivity	1,328	0,229–7,713	0,752	0,71	0,120–4,216	0,706

Abbreviations: *DFS* Disease free survival; *OS* Overall survival; *HR* Hazard ratio; *CI* Confidence interval.

## Discussion

The expression of CTAs has been described in several malignant tumors [5,6,25-27] CTAs have been identified in melanomas, non-small cell lung and pancreatic cancer, serous ovarian cancer, hepatocellular carcinomas, multiple myelomas as well as in breast cancer [17]. The CTA expression frequency in breast cancer varies in the literature reaching up to 88% [14]. However, the reproducibility of the studies suffers in terms of standardization regarding tumor specimen (primary tumors or metastases), methodology (RT-PCR, Western-blot or immunohistochemistry), and the evaluation of the IHC-staining.

Using a broad spectrum of diverse mAbs, we found a total percentage for the presence of any CTA of 37.6%, which is in accordance with most of the existing reports [14]. However, our cohort included solely tissues from primary tumors and in contrast to other reports we valued all cases with weak staining as negative. One study [4] found a positivity of 47% in primary breast tumors, however, including also the tumors with 1–2% positive stained cells. The same authors reported a significant higher percentage of CTA expression in metastatic tumors (66%). These findings fit very well into the tumorbiological context of this gene familiy and reflect their potential role as tumor associated antigens in tumor progression. The antibodies tested in our study revealed the same distribution pattern, concerning isolated cells or groups of cells, differing, however, in the degree of expression. MAGE-A1 and NY-ESO-1 were detected at higher frequency and we recorded neither a significant coexpression nor a mutual exclusion of the various CTAs, in accordance with the literature. However, we could not confirm the reported higher expression of CTAs in estrogen receptor negative cases. Our findings of a clear restriction of CTA expression to grade 2 and 3 cancers is in concordance with other studies [28], however, the small number of grade 1 tumors did not allow us to perform a reliable statistical analysis in this case.

CTA expression was recently associated to prognosis with an adverse impact in gastrointestinal stromal tumors [25], oral squamous cell carcinomas [29], multiple myelomas [30], and cervical cancers [31]. However, controversial findings were also reported correlating CTAs with a less aggressive tumor behaviour [32,33]. Our findings demonstrate a clear association for CTA expression and prognosis. Of all the antibodies tested in our study, M3H67 reactivity seems to exhibit the strongest prognostic impact for the course of breast cancer. MAGE-A proteins bind to KAP1 which is a repressor of p53 and suppress apoptosis in MAGE-A expressing cell lines [34]. Small interfering RNA (siRNA) suppression of MAGE genes leads to increased p53 expression and increased apoptosis in melanoma cell lines [34], thus the overexpression of MAGE proteins in breast cancer could also protect malignant cells from programmed cell death. For MAGE-A3, specifically, a reverse correlation is shown in pituitary tumors between tumor supressive FGFR2 and MAGE-A3 mRNA expression [35], where siRNA down-regulation of MAGE-A3 results in p53 promoter activation and reduced cell proliferation. GAGE proteins seem to have a similar function, since its transfection can render cells resistant against interferon-gamma or death receptor Fas/CD95/APO-1 induced apoptosis [36]. Clinically, overexpression of these proteins seems, indeed, to correlate with adverse prognosis. Due to the fact, that CTAs are relatively widely expressed, this marker could give the additional information for a substantial proportion of breast cancer patients. 57B reactivity had a prognostic relevance in univariate analysis, however, it could not be validated as an independent prognostic factor in the multivariate approach. This limitation might be due to the relatively small number of cases available for statistical analyses. 57B immunoreactivity has been previously associated with poor prognosis in cholangiocarcinoma [37]. Additionally, M3H67 immunoreactivity, as a marker for MAGE-A expression, mainly MAGE-A3, was found to be associated with poor prognosis in gastrointestinal stromal tumors [38]. Moreover MAGE-A3 expression detected with RT-PCR had an adverse prognostic effect in non-small-cell lung cancers [39]. Most previous studies also recognized an adverse correlation of MAGE A family antigens either to the survival or indirectly to established prognostic factors [4,40], with a unique report of MAGE-A4 to be a favourable prognostic factor [33].

In the development of vaccines against breast cancer two major target antigen groups have been proposed: CTAs because of their unique expression pattern in tumor, but not in normal tissue and the breast differentiation antigens. Although our lack of knowledge about the biological function of CTAs complicates their utilisation, the use of CTAs as targets for the vaccination of breast cancer has been under debate widely the last years [41].

The exact biological function of NY-ESO-1 remains unknown. However recent experiments indicate a possible relevance of NY-ESO-1 expression for DNA-methylation. [42]. The frequent expression of NY-ESO-1 in our cohort could play a potential role in the application of additional immunological therapies in breast cancer, since it has been demonstrated that NY-ESO-1 can elicit strong CD8 and CD4 T-cell response in seropositive patients [15,43,44]. Therefore it has been target of several vaccination efforts in the past [11]. In vivo the T-cell responses against tumor-associated antigens seem to improve the prognosis in hepatocellular carcinoma [45]. However, suppression of the immune response via regulatory T-cells has also been described [46]. Several clinical trials [47] have been performed on vaccines targeting breast cancer and two new trials are now recruiting for the use of CTAs as targets. A recent study [16] has showed that CTA expression is more frequent in triple negative breast cancer. This is of particular interest, since our conventional adjuvant therapeutic possibilities in this subgroup of breast cancer are limited.

An important consideration when conducting immunohistochemical studies on the MAGE-A family proteins is their high homology. Cross-reactivity of antibodies to MAGE-A CTAs cannot be ruled out. At this point solely mAb MA454 to MAGE-A1 can be regarded as truly specific for a particular MAGE-A antigen. Attempts to generate reagents to other MAGE-A family members such as MAGE-A3, the most prevalent MAGE-A antigen on a molecular level, have rendered mixed results. This is best exemplified by mAb 57B, which was originally generated as a MAGE-A3 reagent [20]. Subsequent analysis indicated reactivity with several MAGE-A family members [19]. More recent data indicate reactivity of mAb 57B to MAGE-A4 [48]. The same applies to mAb M3H67 which was originally generated to MAGE-A3 but is now considered reactive with several members of the MAGE-A family (unpublished data). However this does not necessarily negatively impact the prognostic value of immunohistochemistry, but it complicates the identification of the best target for cancer immunotherapy. Also we expected that positivity for mAb 6C1, which reacts with several MAGE-A antigens, would be more frequent and comparable to the other anti-MAGE-A reagents. However, in our series this was not the case. This could be based on different affinities of the various reagents for similar antigens generating incongruent staining patters in spite of overlapping specifity patterns.

NY-BR-1 can be identified at the protein level in physiological as well as cancerous breast tissue [49] although recently it has been also described in a vulvar lesion [50]. The function of NY-BR-1 in vivo has not yet been clarified. Bioinformatics analyses showing a DNA-binding site followed by a leucine zipper motif suggest that this molecule acts as a transcription factor. Because of five tandem ankyrin repeats NY-BR-1 could also have a role in protein-protein interactions [49]. Our data suggest that NY-BR-1 is strongly expressed in a great proportion of primary breast cancers (46.6%). This frequent expression of NY-BR-1 has been previously described [12]. Humoral immune response against endogenous NY-BR-1 has been confirmed by detecting the spontaneous NY-BR-1 directed antibody responses in breast cancer patients, tested positive for NY-BR-1 by RT-PCR [51]. Additionally two HLA-A2 restricted peptide epitopes for NY-BR-1 that were recognized by CD8+ T cells derived from breast cancer patients have been defined [52]. Due to the restricted expression pattern, combined with the wide expression in tumors, NY-BR-1 seems to be an ideal potential target for innovative immunotherapeutic approaches of breast cancer because of the more frequent expression in comparison to HER2/neu, the current reference target for cancer immunotherapy. This approach exerts even more potential since we could not confirm a recently reported correlation between NY-BR-1 and HER2/neu expression [53].

Our analyses did not show any significant co-expression of NY-BR-1 with the CT-antigens, neither a mutual exclusion. Since M3H67 reactivity was associated with tumor progression while NY-BR-1 represents a differentiation antigen it might be possible that these tumors with a high M3H67 reactivity and simultaneous absence of NY-BR-1 expression behave in a tumorbiological aggressive fashion. In our cohort, we observed six such cases with an indeed high mortality rate (50%), however the number of cases was too small to extract any further conclusions.

In total 60.3% of our patients were positive for either CT-antigens or NY-BR-1 or both. Theoretically this could facilitate polyvalent vaccines containing more than one antigen in order to achieve in parallel targeting of a higher percentage of tumor cells in genetically heterogeneous tumors, or vaccines that can be used without prior antigen monitoring. The highly immunogenic potential of CT-antigens combined with immune response adjuvants [11] is not yet fully explored but appears promising.

## Conclusions

To our knowledge this study is the largest retrospective analysis of the expression and prognostic role of numerous CT-antigens and NY-BR-1 in breast cancer. Despite the above limitations we believe that our results underline the emerging role of the above group of genes for prognosis and therapeutical approaches in breast cancer in the future. Especially mAb M3H67 reactivity, probably reflecting presence of several MAGE-A antigens was proven as a strong independent prognostic factor. The relatively small number of patients may have concealed other important clinical correlations that appeared only as trends. Therefore a prospective study with a much greater number of patients and the possibility of stratification according to primary and adjuvant therapy is imperatively needed.

## Competing interests

All authors declare to have no financial or non-financial competing interests. There is no funding source to be disclosed.

## Authors’ contributions

DB participated in the array analysis, performed with DD the statistical analysis and drafted the manuscript. ES and AzH conceived the study and participated in its design and coordination. AzH additionally performed the pathological evaluation of the specimens and participated in the array analysis. SM was responsible for the recruitment of the patients in the study and obtained the informed consent. MJ and SM generated the tissue microarrays. AJ provided the monoclonal antibodies and carried out the immunochistochemical staining. MH contributed to the evaluation of the results. GG coordinated the team and made the final corrections. All authors read and approved the final manuscript.

## Pre-publication history

The pre-publication history for this paper can be accessed here:

http://www.biomedcentral.com/1471-2407/13/271/prepub
